# Effect of Topical Vancomycin on Surgical Site Infections in Ankle Fractures: A Randomized, Double-Blind, Controlled Trial

**DOI:** 10.7759/cureus.63694

**Published:** 2024-07-02

**Authors:** Carlos A Acosta-Olivo, Alejandro Hernández-Alejo, Anna K Rangel-Alanís, Jorge A Elizondo-Rodríguez, Héctor M Zertuche-Garza, Yadira A Tamez-Mata, Víctor M Peña-Martínez, Mario Simental-Mendía

**Affiliations:** 1 Orthopedic Trauma Service, School of Medicine, Dr. José Eleuterio González" University Hospital, Universidad Autónoma de Nuevo León, Monterrey, MEX

**Keywords:** internal fixation, open fracture reduction, randomized clinical trial, vancomycin powder, ankle fractures

## Abstract

Background

Applying topical vancomycin has shown a decrease in the likelihood of surgical site infections (SSIs) in surgeries linked to a heightened risk of severe and resistant infections. Nevertheless, the effectiveness of this prophylactic approach has not been assessed in open ankle surgeries with internal fixation.

Objective

This study aimed to assess whether topical vancomycin diminishes the risk of SSI in patients with ankle fractures undergoing open reduction with internal fixation.

Methods

A randomized, controlled, double-blind clinical trial was carried out. Patients were divided into two groups in a 1:1 ratio. The control group received the standard prophylactic treatment with IV cephalothin 1 g, while the intervention group was administered topical vancomycin (1 g) in addition to the standard prophylactic treatment. The main outcomes were the SSI rates at 14 days, 28 days, and three months post-surgery, based on relevant clinical signs and laboratory tests.

Results

One hundred thirty-two patients were randomized (51.2% female), with 66 subjects included in each intervention arm. A total of 97.7% of them completed the study. Both groups were homogeneous in baseline characteristics. There were two SSIs in both the vancomycin group (3.3%) and the control group (3.5%), with no statistical differences (p = 0.945). The microorganisms isolated as causal agents were *Staphylococcus aureus* and *Acinetobacter baumannii*. By the three-month follow-up, no infections were noted in both intervention groups.

Conclusion

These results indicate that the topical administration of vancomycin may not represent an advantage in preventing SSI in ankle fractures requiring open reduction with internal fixation at the three-month postoperative stage.

## Introduction

Ankle fractures are the fourth most frequent type of fracture in adults, behind hip, wrist, and hand fractures [1]. They occur at an annual incidence of 169 per 100,000 people, with an average age of 41 years in the general population, and women being more commonly affected [2]. Falls and traffic accidents are the primary causes [3], with up to 71% of ankle fractures resulting from low-energy mechanisms, particularly in elderly individuals with comorbidities [4].

The selection of the most suitable treatment approach hinges on factors such as the injury's mechanism and an accurate assessment of the lesion, including any associated damage to the soft tissues [2]. The treatment objective is to enable the patient to bear their full body weight on the affected joint without experiencing pain and to avert any lasting damage [3].

According to the Denis Weber classification system [5], an isolated type A fracture of the lateral malleolus should be considered for open reduction and internal fixation (ORIF) only in cases where there are displaced fragments and/or joint involvement. Patients with Type B and C fractures necessitate ORIF management [3]. Therapeutic intervention with ORIF has shown improved functional capacity, reduced edema, and a greater range of ankle motion compared to closed treatment [6]. However, surgical site infection (SSI) stands out as the most prevalent hospital-acquired infection linked to ORIF, having a detrimental impact on both the formation of a solid bone union and the functional recuperation of the ankle [4].

In orthopedic trauma surgery, IV antibiotics have been advocated to have a positive impact on preventing infections [7]. Nonetheless, the local application of antibiotics has garnered attention in recent years due to numerous potential benefits, especially in spine surgery [8,9].

The use of topical vancomycin is suggested for patients undergoing surgeries associated with a high risk of severe and resistant infections, such as spinal, thoracic, and cranial procedures, which has been demonstrated to reduce the risk of SSI [10,11]. However, this prophylaxis method has not been evaluated in patients undergoing open ankle surgery with internal fixation. Hence, the objective of this study was to determine whether the use of topical vancomycin reduces the risk of SSI in patients with ankle fractures who have undergone open reduction with internal fixation.

## Materials and methods

Study design and oversight

A randomized, controlled, double-blind clinical trial was designed in which patients with ankle fractures undergoing surgical treatment with ORIF were recruited. Informed consent was obtained from all individual participants included in the study. All procedures were performed following the ethical standards of the institutional research committee (Research Ethics Committee, reference number: OR20-00007) and the 1964 Helsinki Declaration and its later amendments or comparable ethical standards. The study protocol was registered in the ClinicalTrials.gov database (NCT05363462).

Patient selection

The inclusion criteria comprised patients over 18 years of age, with a history of a closed fracture regardless of the injury mechanism and classification, requiring open reduction surgery with internal fixation for the fracture. Exclusion criteria included patients with open fractures, the presence of immunodeficiency, hypersensitivity or allergy to vancomycin, ankle surgery within the last six months, immunosuppressant use, pregnancy, use of steroids, and antibiotic therapy one week prior to admission. Patients were screened for eligibility at the outpatient clinic.

Randomization and treatment groups

The randomization sequence was generated by a dedicated free-access online tool (https://www.randomizer.org/) by one of the researchers. Participants were assigned to the control and intervention groups in a 1:1 ratio at the preoperative site by another crew member. Allocation concealment was performed using previously sealed envelopes following the randomization sequence by an independent researcher. The randomization list was kept sealed until the completion of the study. The patients and the outcome evaluators were blinded to the allocated intervention. The control group received the standard prophylactic treatment consisting of intravenous administration of cefalotin 1 g one hour before the procedure, followed by intravenous administration of cefalotin 1 g every 8 hours for the next 24 hours during the hospital stay. In addition to the standard prophylactic treatment, the intervention group was administered topical vancomycin powder (1 g) directly to the surgical wound before closure, once the fixation was completed.

Outcomes and follow-up

The presence of SSI as the primary outcome was evaluated at 14 days, 28 days, and 3 months post-procedure, according to the criteria established by the CDC [12]. Relevant clinical signs indicative of infection such as redness, purulent drainage, incision opening, fever, pain, warmth, swelling, or tenderness were intentionally identified. If an infection was suspected, laboratory tests including a complete blood count, erythrocyte sedimentation rate, and C-reactive protein were conducted. In the case of altered parameters, a microbiological culture was requested to determine the causative agent, along with an antibiogram to assess its susceptibility. Patients who showed signs of superficial infection were treated with oral antibiotics for two weeks, with daily cleansing and sterile dressing, while patients with deep infections underwent surgical debridement and IV antibiotics.

Data collection

Different variables were systematically collected to ensure a comprehensive assessment of the diverse factors influencing ankle fractures in the studied population. Key variables included the participants' age (years) at the time of the study; sex (female and male); fracture classification, subcategorized into Weber B, Weber C, and medial malleolar associated fractures to provide a detailed classification of ankle fractures; fracture site, documented to distinguish between occurrences in the right and left ankles; dislocation; and bleeding, which was further divided into two categories: <30 mL and ≥30 mL. Additionally, participants' comorbidities were documented to understand the potential impact of pre-existing health conditions on ankle fractures.

Statistical analysis

Descriptive statistics, including frequencies and measures of central tendency, were employed as appropriate. Risks and differences were assessed using parametric and non-parametric tests based on variable characteristics. The Kolmogorov-Smirnov test was utilized to evaluate data normality; results indicated that the sample distribution was normal in both study groups (vancomycin, D(66) = 0.095, p = 0.20; control, D(66) = 0.075, p = 0.20). Student's t-test was applied to compare parametric variables. Inferential statistical tests were performed using the chi-square test and Fisher's exact test when necessary. A statistical significance threshold of less than 0.05 was considered. Data analysis was conducted using SPSS v25 software for Windows.

Sample size calculation

Based on previous data [4], a two-proportion estimation formula was employed with the primary objective of determining whether the use of topical vancomycin reduces the risk of SSI in patients with ankle fractures who have undergone ORIF. A 95% confidence level and 80% power, with a one-sided significance level of 5%, resulted in a minimum of 130 study subjects.

## Results

Study flow of participants

**Figure 1:**
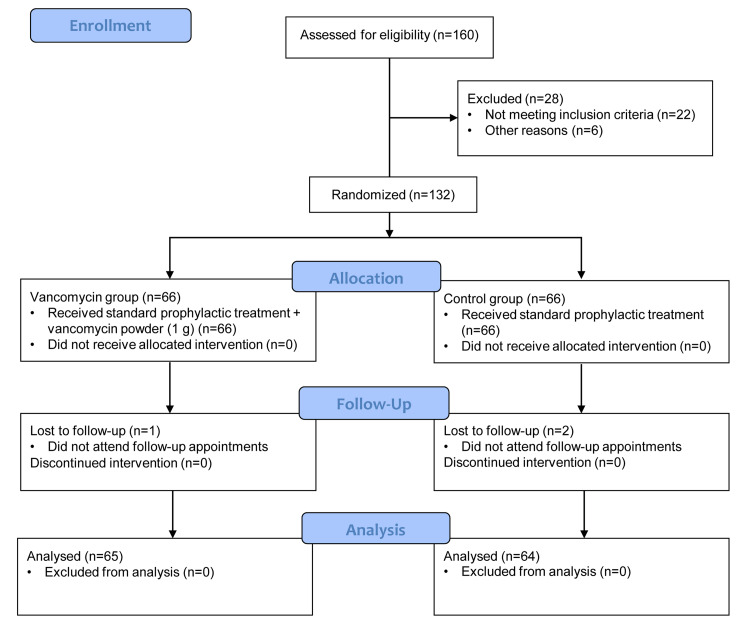
Enrollment process of study participants (CONSORT flow diagram). CONSORT: Consolidated Standards of Reporting Trials.

A total of 160 patients were assessed for eligibility. During the selection process, 28 patients were excluded mainly because they did not meet the inclusion criteria (n = 22). Afterward, 132 subjects meeting the inclusion criteria were allocated to each intervention arm. The topical vancomycin group included 66 patients, while 66 participants were included in the control group. One patient was lost from the vancomycin group, and two patients were lost from the control group (Figure 1).

Patient demographics

Regarding patient demographics, 51.2% were female and 48.8% were male, with an average age of 38.8 ± 13.8 years. A total of 47.3% of the patients presented with a lesion in the right limb, and 52.7% in the left limb. The predominant fracture type according to the Weber classification was Weber B in 71.3% of the patients. Upon conducting comparisons, no statistically significant differences were observed between the vancomycin and control groups in terms of age, sex, fracture type, fracture side, and presence of dislocation (Table 1). Therefore, the groups were homogeneous in their baseline characteristics.

**Table 1:** Baseline characteristics of the patients. †Student’s t-test; ‡Fisher’s exact test; §Chi-square test.

Characteristics	Vancomycin group (n = 65)	Control group (n = 64)	P-value
Age (years), mean ± SD	37.7 ± 14.6	40.1 ± 13.0	0.329 (t=0.9801) †
Sex, n (%)			
Female	33 (50.8)	33 (51.5)	0.928‡
Male	32 (49.2)	31 (48.5)	
Classification, n (%)			
Weber B	43 (66.2)	49 (76.6)	0.395§
Weber C	16 (24.6)	10 (15.6)	
Medial malleolar	6 (9.2)	5 (7.8)	
Fracture side, n (%)			
Right	35 (53.8)	26 (40.6)	0.160‡
Left	30 (46.2)	38 (59.4)	
Dislocation, n (%)	17 (26.2)	13 (20.3)	

Effect of vancomycin on SSI

The results of the comparison between the experimental and control groups regarding the presence of SSI are presented in Table 2. At 14 days post-surgery, there were 2 cases (3.3%) in the vancomycin group and 2 cases (3.5%) in the control group, with no statistically significant difference observed (p = 0.945). By day 28, the vancomycin group reported no infections (0.0%), while one case remained infected in the control group (1.7%; p = 0.319). Interestingly, at the three-month postoperative mark, both groups exhibited no infections. The differences in the SSI rate development between the groups were not significantly different at the specified time intervals. The microorganisms isolated as causal agents were *S. aureus* and *Acinetobacter baumannii* in the vancomycin group and only *S. aureus* in the control group. Additionally, parameters such as bleeding, duration of surgery, or patient comorbidities did not appear to influence the results (Table 3). No vancomycin-related adverse events were recorded.

**Table 2:** Comparison of surgical site infection incidence between groups. †Fisher's exact test.

Surgical site infection	Vancomycin group, n (%)	Control group, n (%)	P-value
14 days	2 (3.3)	2 (3.5)	0.600†
28 days	0 (0)	1 (1.7)	
Three months	0 (0)	0 (0)	-

**Table 3:** Secondary outcomes. DM: Diabetes mellitus; AHT: Arterial hypertension. †Fisher’s exact test; ‡Student’s t-test; §Chi-square test.

Outcome	Vancomycin group (n = 65)	Control group (n = 64)	P-value
Bleeding, n (%)			
Bleeding <30 mL	43 (66.1)	41 (64.1)	0.803†
Bleeding ≥30 mL	22 (33.9)	23 (35.9)	
Duration of surgery, (h)	2.5 ± 1.8	2.4 ± 1.5	0.751 (t=3177)‡
Comorbidities, n (%)	16 (24.6)	11 (17.2)	
DM	4	6	0.121§
AHT	6	0	
DM+AHT	2	2	
Others	4	3	

## Discussion

We conducted a randomized, controlled, double-blind clinical trial focusing on patients with ankle fractures undergoing surgical treatment with ORIF. The study compared two groups: one received standard prophylactic treatment with cephalothin, while the other received additional topical vancomycin at the surgical site. The primary outcome was the presence of postoperative SSI, which was below 4.0% in both study groups with no significant differences between them. At both the two-week and four-week postoperative assessments, no statistically significant differences were observed in infection rates between the vancomycin group and the control group. Notably, both groups showed no infections during the three-month postoperative period.

Emerging evidence suggests that topical vancomycin reduces SSI mainly in spine surgeries and tibial fractures [9,11,13,14]. However, conclusive evidence is limited due to conflicting results and the lack of level-I studies. Regarding the use of topical vancomycin in ankle fractures managed with ORIF, reports are sparse. This is a topic of relevance since SSIs are one of the most severe complications due to their potential to significantly worsen patient outcomes [15]. An estimated incidence of SSI in surgically managed ankle fractures ranges from 4.37% to 4.0% [4,16]. Lachman JR et al. reported that superficial infections may represent 4.1%, while deep infections requiring surgical debridement may account for 0.7%; with no relation to the use of preoperative or postoperative intravenous or oral antibiotics [16]. Our results showed similar SSI rates of 3.3% and 3.5% after additional topical vancomycin and conventional antibiotic therapy, respectively. Again, information on ankle SSIs remains limited and controversial due to small sample sizes and studies not designed for this specific purpose. As far as we know, this is the first RCT to deliver results on the use of topical vancomycin in surgically managed ankle fractures.

SSIs are categorized by the CDC as superficial and deep infections. Superficial infections occur within 30 days after a procedure, involving the skin and subcutaneous tissue of the incision. Deep infections occur within 30 days if no implant is left, or up to one year if an implant is present and the infection relates to the implant. These involve deep tissues like fascia and muscle [12]. The presence of purulent drainage, dehiscence, fever, pain, or tenderness could indicate a SSI even without a positive microbiological culture test [12]. The four cases of SSI in our study were superficial and occurred within the first 14 days post-surgery. Only one infection remained active at 28 days; thereafter, all infections were resolved.

A series of recent studies have investigated the efficacy of topical vancomycin in preventing SSI, offering encouraging results related to tibial plateau fractures [13], spine surgeries [17], and pelvic and acetabular surgeries [10]. In contrast, our study assessed infection presence between groups up to three months in closed ankle fractures with no statistically significant differences observed in the evaluations conducted at two weeks, four weeks, and three months. Similar results have been pointed out by a randomized controlled trial of closed fractures treated with ORIF, indicating that topical vancomycin did not represent an advantage in reducing the incidence of infections [18]. A potential explanation for the differences observed in the infection rate reduction may rely upon the difference between intrinsic infection incidences. Unlike ankle fractures managed with ORIF [4], spine surgeries have greater post-surgical infection rates which can be up to 20% [19,20].

Numerous risk factors have been recognized in association with SSI following ORIF for an ankle fracture. These factors include open wounds, advanced age, incisions graded between II and IV according to the surgical wound classification, injuries resulting from high-energy incidents, the experience level of the surgeon, higher BMI, a medical history of chronic heart disease, a previous allergic history, and preoperative neutrophil counts exceeding 75% [4]. Thangarajah T et al. reported in their retrospective study that the most common conditions among patients with SSI in ankle fractures treated with ORIF were hypertension, asthma, diabetes, and epilepsy [21]. In our study, 50% of patients who presented with SSI had diabetes mellitus as a comorbidity (one in each intervention group), the other half did not have any relevant clinical-pathological history, and all subjects were male.

The most common causative agents of SSI often include bacteria such as *Staphylococcus aureus*, Staphylococcus coagulase-negative, or Enterococci, which tend to be multidrug-resistant to commonly used antibiotics; additionally, many of these SSIs have shown to be polymicrobial [20,22]. In this regard, the VANCO Study, a randomized controlled trial conducted across multiple centers, compared the efficacy of local antibiotic therapy using vancomycin powder versus no powder in preventing SSI in patients with high-risk tibia fractures. The trial results indicated a 50% reduction in gram-positive SSI with the localized application of vancomycin powder (relative risk: 0.49, 95% CI, 0.27-0.88, p = 0.02) [23]. An early study also showed an elevated risk of gram-negative and polymicrobial infections associated with the use of vancomycin powder in spine surgeries [14]. Wang H et al. reported in a similar study a higher incidence of infections related to tibial plateau fractures caused by Gram-positive microorganisms in the control group (12/128 subjects) compared to the group where vancomycin was applied (1/105 subjects) [13]. These findings align with the results of our study, where the primary causative agents of SSI were *S. aureus* (Gram-positive) and *Acinetobacter baumannii *(Gram-negative) in the group that utilized vancomycin. In contrast, the control group identified only *S. aureus* as the causative agent.

Within the limitations of our study, there was a lack of follow-up beyond the third month. Nevertheless, by the three-month appointment, all infections were resolved, and no new infections were recorded. Additionally, there was a risk of inadequate control over the presence of contamination by resistant pathogens. Despite having patient history records in this study, potential confounding variables such as the presence of comorbidities increasing the risk of infection and post-discharge home care were not evaluated. However, the homogeneity of the comparison groups strengthens the reliability of our findings. Due to the relatively low SSI rate obtained, a larger sample would be required to confirm the findings. This study sets a precedent for further research.

## Conclusions

The topical application of vancomycin did not significantly impact the incidence of SSI compared to the control group in patients with ankle fractures at the three-month postoperative mark. These results suggest that topical vancomycin may not provide substantial benefits in preventing SSIs in this specific patient population. However, further research with larger sample sizes and longer follow-up periods is warranted to confirm these findings and explore potential nuances in the effectiveness of topical vancomycin in different clinical scenarios.
